# Oridonin alters the expression profiles of MicroRNAs in BxPC-3 human pancreatic cancer cells

**DOI:** 10.1186/s12906-015-0640-5

**Published:** 2015-04-14

**Authors:** Zhifang Gui, Shuquan Li, Xing Liu, Bin Xu, Jian Xu

**Affiliations:** Medical Technology College, Zhejiang Chinese Medical University, Binwen Road, Binjiang District, Hangzhou, 310053 Zhejiang Province China; School of Medicine, Jinggangshan University, Ji’an, 343000 China; Department of General Surgery, Sir Run Run Shaw Hospital, School of Medicine, Zhejiang University, Hangzhou, 310016 China

**Keywords:** Oridonin, miRNA, microarray, BxPC-3 pancreatic cancer cell

## Abstract

**Background:**

Oridonin, an ingredient used in traditional Chinese medicine, has been demonstrated to play an important role in antitumour effects, but the mechanism underlying its antitumour properties is still not clear.

**Methods:**

To verify the anti-cancer effects of oridonin via a miRNA-dependent mechanism, comprehensive miRNA expression profiling of oridonin-treated BxPC-3 human pancreatic cancer cells was performed using a miRNA microarray assay based on Sanger miR-Base Release 20, followed by a validation using real-time PCR. MicroRNA target prediction and Gene Ontology and KEGG pathway analysis were performed to investigate possible pathways involved.

**Results:**

The results showed that 105 miRNAs were significantly differentially expressed (signal reading >500, p ≤ 0.01, |Log2-value| ≥1) in oridonin-treated BxPC-3 human pancreatic cancer cells.

**Conclusions:**

Our data indicates that oridonin inhibits BxPC-3 cells probably through regulating the expression of miRNAs. Interruption of miRNA profiling may provide new therapeutic methods for the clinical treatment of pancreatic cancer.

## Background

Oridonin, a natural ent-kaurane diterpenoid compound, is isolated from the Chinese medicinal herb *Rabdosia rubescens* as well as other plants, such as Isodon trichocarpus and Isodon shikokianus. Oridonin has many physiological and pharmacological effects, including anti-inflammation, anti-bacterial and anti-tumour effects, and shows no obvious side effects when used for the treatment of various human diseases. Concerning anti-tumour effects, previous studies have reported that oridonin can induce cell growth inhibition, promote apoptosis and inhibit migration and invasion in many cancers [1-3]. Nevertheless, the mechanisms underlying the antitumour activity of oridonin have not been completely delineated.

MicroRNAs (miRNAs) are a novel class of non-coding RNAs with lengths of 17–25 nucleotides (nt) that can regulate gene expression in eukaryotic organisms by pairing with target mRNAs to repress translation or cause degradation of multiple target mRNAs [4]. Recent studies have shown that miRNAs play crucial roles in many biological processes, such as development, cell growth, differentiation, apodosis and even tumouriogenesis [5,6]. Furthermore, miRNAs can function both as tumour suppressors and oncogenes and might be a potential therapeutic target in cancer. Recent publications have shown that correcting abnormal miRNAs in tumours can inhibit the function of the target mRNA in vivo in a mouse model [7,8].

Traditional Chinese medicines have become a popular topic in relation to their potential anti-tumour properties. However, there are no available reports on oridonin which inhibits pancreatic cancer via miRNA regulation. In this study, we establish a sensitive microarray chip for miRNA expression profiling in BxPC-3 pancreatic cancer cells treated with oridonin to verify our hypothesis that oridonin alters the miRNA expression profile in pancreatic cancer, and we show that miRNAs have potential applications in the future clinical treatment of tumours.

## Methods

### Cell culture

The BxPC-3 human pancreatic cancer cell line was provided by the Institute of Biochemistry and Cell Biology, Shanghai Institute of Biological Sciences, Chinese Academy of Sciences (ATCC® CRL1687™). The cells were cultured in RPMI 1640 (GIBCO, NY, United States) culture medium containing 10% foetal bovine serum (FBS, Gibco), 300 mg/L glutamine, 100 U/mL penicillin and 100 μg/mL streptomycin in an incubator with 5% CO_2_ at 37°C. Cells in logarithmic growth phase were seeded in 60 mm dishes at a density of 4 × 10^4^ cell/cm^2^ and incubated overnight. One group of these cells was subsequently treated with 87.8 μM oridonin (Gracia Chemical Technology Company, LTD, 98% purity, HPLC) dissolved in DMSO (final DMSO concentration in growth media is 0.1%), and another was used as a blank control group cultured in medium containing 0.1% DMSO for 24 hours. At least 3 independent experiments were performed.

### RNA isolation and miRNA microarray

After 24 hours of treatment, total RNA (containing small RNAs) was extracted using the TRIzol LS reagent (Invitrogen Life Technologies) following the manufacturer’s protocol. The microarray assay (μParafloTM MicroRNA Microarray Assay) was performed by a service provider (LC Sciences), including quality control, labelling, chip hybridisation, signal amplification image acquisition and microarray data analysis. Hybridisation was performed overnight on a μParaflo microfluidic chip using a micro-circulation pump (Atactic Technologies) [9]. On the microfluidic chip, each detection probe consisted of a chemically modified nucleotide coding segment complementary to target microRNA (from miRBase, http://www.mirbase.org/) or other RNA (control or customer defined sequences) and a spacer segment of polyethylene glycol to extend the coding segment away from the substrate. The detection probes were made by in situ synthesis using PGR (photogenerated reagent) chemistry. The hybridization melting temperatures were balanced by chemical modifications of the detection probes. Hybridization used 100 L 6xSSPE buffer (0.90 M NaCl, 60 mM Na_2_HPO_4_, 6 mM EDTA, pH 6.8) containing 25% formamide at 34°C. After RNA hybridization, tag-conjugating Cy3 dye were circulated through the microfluidic chip for dye staining. Fluorescence images were collected using a laser scanner (GenePix 4000B, Molecular Device) and digitized using Array-Pro image analysis software (Media Cybernetics). The data were analysed by first subtracting the background and then normalising the signals using a LOWESS filter (locally weighted regression). Then, the ratio of detected signals showing a log2 fold change [log2 (oridonin/control)] was used to define differentially expressed miRNAs, and Student’s t-test was employed to calculate *P* values.

### MiRNA target prediction and Gene Ontology and KEGG pathway analysis

The prediction of miRNA targets was performed using the online software TargetScan (http://www.targetscan.org/), PicTar (http://pictar.mdc-berlin.de/cgi-bin/new_PicTar_vertebrate.cgi) and miRanda (http://www.microrna.org/microrna/home.do). The intersection of the results from these three types of software was taken as the final target genes of significantly differentially expressed miRNAs. Then, the target genes were analysed in terms of the annotation of their Gene Ontology (GO) categories and Kyoto Encyclopedia of Genes and Genomes (KEGG) pathways using Fisher’s exact test.

### Reverse-transcription and Quantitative Real-time PCR

To validate the microarray data, total RNA from the same preparation used for microarray analysis was reverse-transcribed to cDNA in a Mycycler™ Thermal Cycler (Bio-Rad, USA), and quantitative real-time polymerase chain reaction (qPCR) was performed in a Real-Time PCR Detector (Bio-Rad, USA) using the PrimeScript™ miRNA qPCR Starter Kit Ver.2.0 (TaKaRa, Dalian, China), following the manufacturer’s protocol. Each reaction was performed in a final volume of 25 μl containing 1 μl cDNA, 0.4 μM of each primer and 1× SYBR Premix Ex TaqII. The amplification program was as follows: denaturation at 95°C for 10 sec, followed by 40 cycles of denaturation at 95°C for 5 sec and extension at 60°C for 20 sec, in which fluorescence was obtained. For quantification, RNU6B was used as the internal control, and expression levels of each mature miRNA were normalised using the 2-^△△CT^ method [10]. All assays were performed in triplicate.

### Statistical analysis

A log2 fold change [log2 (oridonin/control)] was used to define differentially expressed miRNAs, and Student’s t-test was employed to calculate P values. The target genes were analysed in terms of the annotation of their Gene Ontology (GO) categories and Kyoto Encyclopedia of Genes and Genomes (KEGG) pathways using Fisher’s exact test. Results of realtime RT-PCR experiments are expressed as means ± standard deviation (SD). Statistical comparisons were performed with the SPSS 17.0 software (Univariate Analysis of Variance) and statistical significance was considered for *P* values lower than 0.05.

## Results

### MiRNA expression was altered in BxPC-3 cells treated with oridonin

To study the responses of miRNAs to oridonin, microarray analysis of miRNA expression in BxPC-3 cells treated with oridonin was compared with the expression of miRNAs in DMSO treated cells. Only miRNAs showing significant expression among the oridonin treatments and their controls are reported (Table 1). As shown in Table 1, 105 reporters presented a strong response (signal reading >500, p ≤ 0.01, |Log2-value^b^| ≥1) and significant regulation. Among these 105 miRNAs, 49 miRNAs were significantly down-regulated, whereas 56 were significantly up-regulated by oridonin. Among them, there are many new miRNAs whose function has been scarcely described in the literature.

**Table 1:** **miRNA regulation of oridonin in BxPC-3 cells**

**Reporter name** ^**a**^	**Log2-value** ^**b**^	**p-Value**	**Reporter name** ^**a**^	**Log2-value** ^**b**^	**p-Value** ^**b**^
hsa-miR-513a-5p	15.70	2.09E-66	hsa-miR-27b-5p	−16.93	0.00E + 00
hsa-miR-3661	12.73	0.00E + 00	hsa-miR-205-3p	−16.63	0.00E + 00
hsa-miR-4470	12.24	0.00E + 00	hsa-miR-4262	−16.47	0.00E + 00
hsa-miR-409-3p	10.68	0.00E + 00	hsa-miR-499a-5p	−16.36	0.00E + 00
hsa-miR-3197	10.32	0.00E + 00	hsa-miR-3934-3p	−16.32	0.00E + 00
hsa-miR-5096	10.29	0.00E + 00	hsa-miR-193b-3p	−7.45	0.00E + 00
hsa-miR-4267	10.13	0.00E + 00	hsa-miR-421	−6.93	0.00E + 00
hsa-miR-466	8.90	0.00E + 00	hsa-miR-10b-3p	−5.74	0.00E + 00
hsa-miR-615-5p	8.80	0.00E + 00	hsa-miR-7641	−5.53	0.00E + 00
hsa-miR-7108-5p	8.13	0.00E + 00	hsa-miR-425-5p	−4.77	0.00E + 00
hsa-miR-6791-5p	7.92	0.00E + 00	hsa-miR-125b-5p	−4.38	0.00E + 00
hsa-miR-1246	6.86	0.00E + 00	hsa-miR-200b-3p	−3.98	0.00E + 00
hsa-miR-6807-5p	6.57	1.38E-43	hsa-miR-3960	−3.95	0.00E + 00
hsa-let-7f-5p	6.41	0.00E + 00	hsa-miR-132-3p	−3.55	0.00E + 00
hsa-miR-1307-3p	5.81	0.00E + 00	hsa-miR-361-5p	−3.34	0.00E + 00
hsa-miR-4514	5.36	1.44E-59	hsa-miR-3178	−3.31	0.00E + 00
hsa-miR-4472	5.22	0.00E + 00	hsa-miR-454-3p	−3.13	0.00E + 00
hsa-miR-6126	5.04	0.00E + 00	hsa-miR-320b	−2.71	0.00E + 00
hsa-miR-6073	4.60	1.24E-43	hsa-miR-455-3p	−2.62	0.00E + 00
hsa-miR-4301	4.26	0.00E + 00	hsa-miR-320e	−2.56	0.00E + 00
hsa-miR-4484	3.79	3.65E-71	hsa-miR-185-5p	−2.47	0.00E + 00
hsa-miR-30c-1-3p	3.65	0.00E + 00	hsa-miR-320c	−2.38	0.00E + 00
hsa-miR-4447	3.59	2.47E-42	hsa-miR-4521	−2.13	0.00E + 00
hsa-miR-7977	3.56	0.00E + 00	hsa-miR-320a	−2.10	0.00E + 00
hsa-miR-5787	3.47	2.93E-51	hsa-miR-193a-3p	−2.09	0.00E + 00
hsa-miR-7150	3.44	2.24E-47	hsa-miR-320d	−2.04	0.00E + 00
hsa-miR-4516	3.16	0.00E + 00	hsa-miR-92b-3p	−1.84	0.00E + 00
hsa-miR-1273 g-3p	3.06	0.00E + 00	hsa-let-7i-5p	−1.81	0.00E + 00
hsa-miR-6090	3.00	0.00E + 00	hsa-miR-183-5p	−1.79	0.00E + 00
hsa-miR-494-3p	2.98	1.06E-72	hsa-miR-365a-3p	−1.59	0.00E + 00
hsa-miR-6786-5p	2.87	1.26E-23	hsa-miR-186-5p	−1.54	0.00E + 00
hsa-miR-6727-5p	2.81	0.00E + 00	hsa-miR-125a-5p	−1.54	0.00E + 00
hsa-miR-98-5p	2.65	0.00E + 00	hsa-miR-151a-3p	−1.43	0.00E + 00
hsa-miR-668-3p	2.64	6.84E-65	hsa-miR-224-5p	−1.42	0.00E + 00
hsa-miR-1275	2.59	1.69E-50	hsa-miR-107	−1.40	0.00E + 00
hsa-miR-6125	2.53	1.03E-49	hsa-miR-93-5p	−1.34	0.00E + 00
hsa-miR-6087	2.50	0.00E + 00	hsa-miR-3609	−1.31	0.00E + 00
hsa-miR-4505	2.31	1.03E-10	sa-miR-103a-3p	−1.28	0.00E + 00
ha-smiR-7110-5p	2.26	3.03E-12	hsa-miR-4286	−1.22	0.00E + 00
hsa-miR-1260a	2.14	1.31E-09	hsa-miR-3607-5p	−1.20	0.00E + 00
hsa-miR-6803-5p	2.13	1.94E-10	hsa-miR-92a-3p	−1.19	0.00E + 00
hsa-miR-29c-3p	2.08	2.01E-13	hsa-miR-429	−1.17	0.00E + 00
hsa-miR-4466	2.04	3.57E-06	hsa-miR-20a-5p	−1.15	0.00E + 00
hsa-miR-1973	1.93	7.49E-09	hsa-miR-424-5p	−1.13	0.00E + 00
hsa-miR-4739	1.86	1.47E-04	hsa-miR-17-5p	−1.09	0.00E + 00
hsa-miR-3196	1.85	1.80E-15	hsa-miR-203a	−1.05	0.00E + 00
hsa-miR-4497	1.76	1.16E-13	hsa-miR-574-3p	−1.04	0.00E + 00
hsa-miR-378 g	1.76	2.93E-03	hsa-miR-378c	−1.03	0.00E + 00
hsa-miR-4459	1.73	5.05E-03	hsa-miR-423-5p	−1.01	0.00E + 00
hsa-miR-3665	1.70	2.46E-11			
hsa-miR-638	1.64	2.41E-04			
hsa-miR-7704	1.63	4.06E-08			
hsa-let-7 g-5p	1.54	1.18E-05			
hsa-let-7e-5p	1.52	3.00E-07			
hsa-miR-4508	1.04	2.30E-08			
hsa-miR-6089	1.03	2.17E-19			
**Reporter name** ^**c**^	**Log2-value** ^**b**^	**p-Value**	**Reporter name** ^**c**^	**Log2-value** ^**b**^	**p-Value**
hsa-miR-328-5p	2.96	3.36E-18	hsa-miR-3943	−16.15	0.00E + 00
hsa-miR-5194	6.18	8.69E-39	hsa-miR-4536-3p	−16.08	0.00E + 00
hsa-miR-6085	2.55	2.40E-12	hsa-miR-1180-3p	−15.86	0.00E + 00
hsa-miR-6880-5p	2.69	1.08E-13	hsa-miR-3188	−15.82	0.00E + 00
hsa-miR-4791	14.87	2.29E-39	hsa-miR-1179	−15.81	0.00E + 00
hsa-miR-6124	3.00	1.28E-16	hsa-miR-3169	−9.50	0.00E + 00
hsa-miR-1233-5p	3.54	1.32E-20	hsa-miR-15b-3p	−7.42	0.00E + 00
hsa-miR-765	2.56	9.53E-10	hsa-miR-301a-3p	−6.71	0.00E + 00
hsa-miR-4463	2.34	1.77E-07	hsa-miR-101-3p	−6.63	0.00E + 00
			hsa-miR-3065-5p	−6.07	0.00E + 00
			hsa-miR-625-5p	−3.97	0.00E + 00
			hsa-miR-24-2-5p	−3.06	0.00E + 00
			hsa-miR-128-3p	−2.86	0.00E + 00
			hsa-miR-4289	−2.65	0.00E + 00
			hsa-miR-155-5p	−2.35	0.00E + 00
			hsa-miR-197-3p	−2.21	0.00E + 00
			hsa-miR-10a-5p	−2.08	0.00E + 00

^a^Transcripts showing strong signals (signal ≥ 500; |Log2-value| ≥ 1).

^b^Oridonin/control.

^c^Transcripts showing weak signals (350 < signal < 500; |Log2-value| ≥ 2).

Previous studies related to miRNA expression in human pancreatic cancer are collected and summarised in Table 2 for comparison and discussion. Results showed that the expression of some miRNAs was changed dramatically after treatment with oridonin, as shown in Table 3 (20 miRNAs, including miR-205, miR-10b, miR-125b, miR-200b, miR-132, miR-320, miR-185, miR-424-5p, and miR-17-5p), which indicated that oridonin may influence BxPC-3 pancreatic cancer cells through regulating miRNAs, though verifying this hypothesis will require further investigation.

**Table 2:** **miRNA expression in pancreatic cancer**

**miRNA**	**Regulation**	**Source**	**Reference**
miR-17-5p	up	pancreatic cancer cell lines ( AsPC-1, KP-1 N, KP-3 and PANC-1 et al.)	[11,12]
miR-10a	up	15 pancreatic cancer cell lines	[12]
miR-210	up	pancreatic cancer patients	[13]
miR-214	up	pancreatic cancer tissues	[14]
miR-15a	down		
miR-107	up	MiaPACA-2 and PANC-1 cells	[15,16]
miR-103	up		
miR-29a	up		
miR-320	up		
miR-375	down	Panc-1, SW1990, BxpC3 and Patu8988	[17]
miR-483-3p	up	pancreatic cancer tissues	[18]
miR-21	up	pancreatic cancer pecimens and 14 pancreatic cancer cell lines	[19,20]
miR-146a	down	Colo357 and Panc-1	[21]
miR-424-5p	up	Human PDAC Tissues and PDAC Cell Lines	[22]
miR-155	up		
miR-221	up		
Let-7	down	Pancreatic ductal adenocarcinoma samples	[23]
miR-126	down	pancreatic tissue samples and cell lines	[24]
miR-132	up	Pancreatic adenocarcinoma (PDAC) tissues	[25]
miR-212	up		
miR-96	down	pancreatic cancer tissues and cell lines	[26]
miR-217	down	PDAC tissues and cell lines	[27]
miR-494*	up	BxPC-3 cell	[28]
miR-140	up		
miR-148a*	up		
miR-200b*	up		
miR-564*	up		
miR-195*	up		
miR-637*	up		
miR-34a	down	MIA PaCa-2 and AsPC-1 cells	[29]
miR-29c	down	normal pancreas and PDAC tissue	[30]
miR-494	down		
miR-615-5p	down	BxPC-3, CFPAC-1, SW1990, PANC-1	[31]
95 miRNA (let-7-family, miR-7, miR-92 and miR-93 et al.)	up	BxPC-3 cell	[16]

*Passenger strand.

**Table 3:** **Differential expression of miRNAs in pancreatic cancer and pancreatic cancer induced by oridonin**

**miRNA**	**Regulation**	^**a**^ **Regulation reported in literature**	**Source in literature**	**Reference**
miR-205	down	up	BxPC-3 cell	[16]
miR-10b	down	up	BxPC-3 cell	[16]
miR-125b	down	up	BxPC-3 cell	[16]
miR-200b	down	up	BxPC-3 cell	[16]
miR-132	down	up	BxPC-3 cell	[16]
miR-320	down	up	MiaPACA-2 and PANC-1 cells	[15]
miR-185	down	up	BxPC-3 cell	[16]
miR-92	down	up	BxPC-3 cell	[16]
miR-183	down	up	BxPC-3 cell	[16]
miR-186	down	up	BxPC-3 cell	[16]
miR-125a	down	up	BxPC-3 cell	[16]
miR-151	down	up	BxPC-3 cell	[16]
miR-224	down	up	BxPC-3 cell	[16]
miR-107	down	up	MiaPACA-2, PANC-1 and BxPC-3 cells	[15,16]
miR-93	down	up	BxPC-3 cell	[16]
miR-103	down	up	MiaPACA-2, PANC-1 and BxPC-3 cells	[15,16]
miR-20a	down	up	BxPC-3 cell	[16]
miR-424-5p	down	up	Human PDAC Tissues and PDAC Cell Lines	[22]
miR-17-5p	down	up	14 pancreatic cancer cell lines (AsPC-1, KP-1 N, KP-3 and PANC-1 et al.)	[11]
miR-203	down	up	BxPC-3 cell	[16]
miR-29c-3p	up	down	normal pancreas and PDAC tissue	[30]
miR-494	up	down		
miR-615-5p	up	down	BxPC-3, CFPAC-1, SW1990 and PANC-1	[31]

^a^Regulation reported in pancreatic cancer tissues/cells compared with normal pancreatic tissues/cells from the literature.

### Target prediction and GO and KEGG pathway analyses

It has been demonstrated that one miRNA could target more than one gene, whereas some genes were targets of more than one miRNA. To predict the target mRNAs of the differentially expressed miRNAs, we performed target prediction for the differentially expressed miRNAs identified in the BxPC-3 cells using three different types of online software: TargetScan, PicTar and miRanda. The intersection of three software’s predictions was taken as the finally potential target genes.

GO and KEGG pathway analyses were performed on the target genes of the significantly differentially expressed miRNAs.

**Figure 1:**
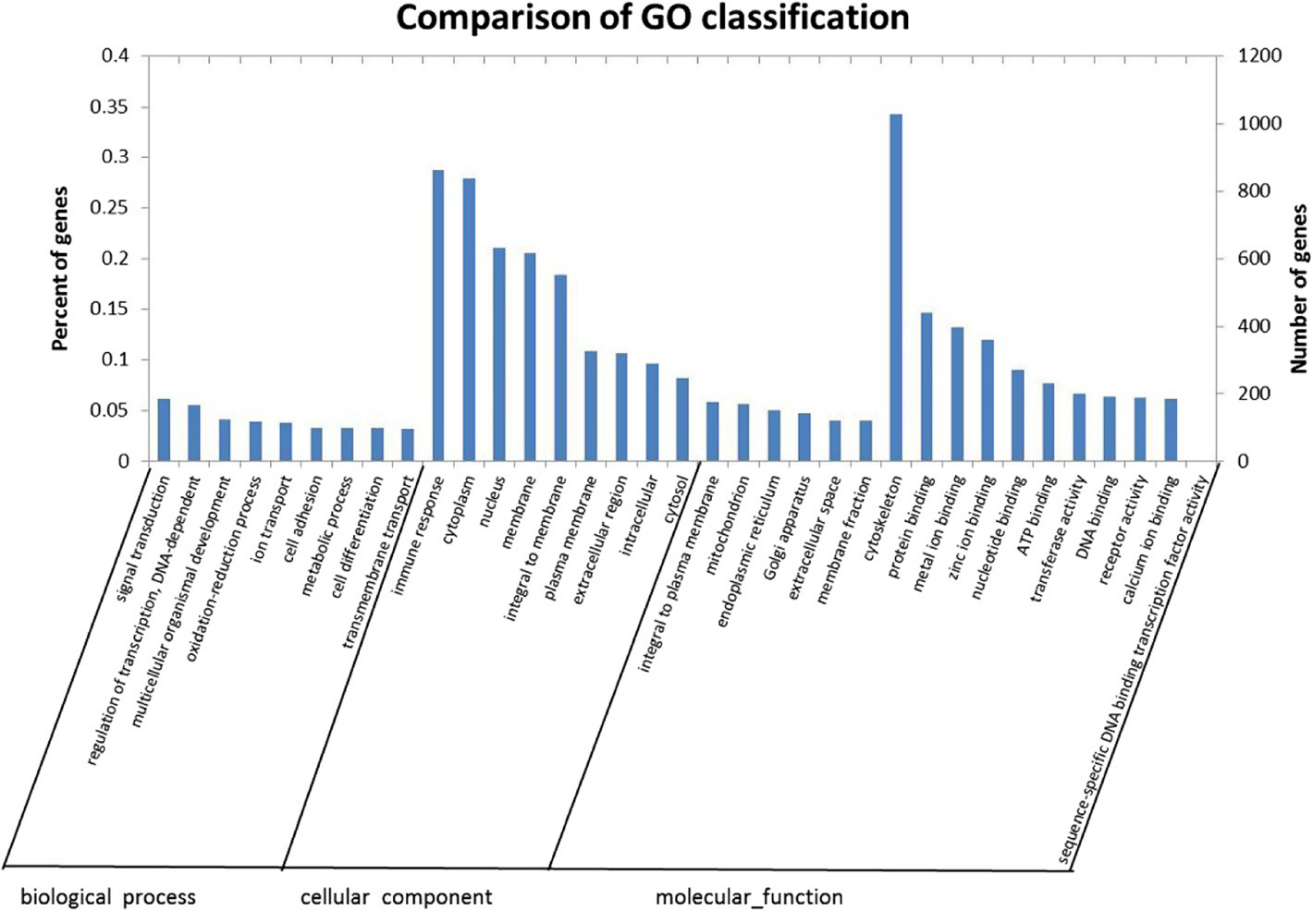
Distribution of GO categories for the predicted target genes of differentially expressed miRNAs identified in BxPC-3 cells treated with oridonin. The left vertical axis represents the percent of genes, the right vertical axis represents the number of genes and the horizontal axis represents the GO category, including biological processes, cellular components and molecular functions.

The enriched GO annotations are shown in Figure 1. The results revealed that the significantly enriched predicted target genes were involved mainly in the following categories: biological processes (e.g., signal transduction, regulation of transcription, DNA-dependent and multicellular organismal development), cellular components (e.g., cytoplasm, nucleus, membrane, integral to membrane and plasma membrane) and molecular functions (protein binding, metal ion binding and zinc ion binding).

The KEGG pathway annotations of all of the target genes of the significantly differentially expressed miRNAs are shown in Table 4 (P ≥ 0.05). KEGG is a major public database of biological pathways, and significant enrichment in KEGG categories can identify differentially expressed genes involved in the main biochemical metabolic pathways and signal transduction pathways. The results presented in Table 4 revealed that the influence of BxPC-3 pancreatic cancer cells by oridonin may be related to neuroactive ligand-receptor interactions, pathways involved in cancer, MAPK signalling pathways, focal adhesion, calcium signalling pathways and other factors, prompting further study on the mechanism of pancreatic cancer inhibition by oridonin.

**Table 4:** **KEGG pathway annotation of the targets of differentially expressed miRNAs identified in BxPC-3 cells treated with oridonin**

**Pathway Id**	**Pathway description**	^**a**^ **S gene number**	^**b**^ **TS gene number**	^**c**^ **B gene number**	^**d**^ **TB gene number**	***P*** **value**
4080	Neuroactive ligand-receptor interaction	176	2158	206	2734	0.00899472
5200	Pathways in cancer	174	2158	204	2734	0.010837484
4010	MAPK signaling pathway	138	2158	155	2734	0.000542141
4510	Focal adhesion	123	2158	141	2734	0.00651097
4020	Calcium signaling pathway	118	2158	132	2734	0.000957212
4144	Endocytosis	92	2158	104	2734	0.00754512
4514	Cell adhesion molecules (CAMs)	79	2158	91	2734	0.03558939
4142	Lysosome	73	2158	79	2734	0.000942336
4640	Hematopoietic cell lineage	70	2158	76	2734	0.001553248
4670	Leukocyte transendothelial migration	69	2158	78	2734	0.0203194
4722	Neurotrophin signaling pathway	67	2158	76	2734	0.026182028
4660	T cell receptor signaling pathway	65	2158	74	2734	0.033536383
5414	Dilated cardiomyopathy	63	2158	68	2734	0.001810577
5410	Hypertrophic cardiomyopathy (HCM)	62	2158	66	2734	0.000696228
4350	TGF-beta signaling pathway	56	2158	62	2734	0.013956825
4512	ECM-receptor interaction	56	2158	63	2734	0.029353732
4912	GnRH signaling pathway	55	2158	62	2734	0.033519609
5215	Prostate cancer	52	2158	56	2734	0.004141376
5412	Arrhythmogenic right ventricular cardiomyopathy (ARVC)	52	2158	56	2734	0.004141376
4730	Long-term depression	47	2158	51	2734	0.009668211
5212	Pancreatic cancer	45	2158	50	2734	0.031740035
4720	Long-term potentiation	44	2158	48	2734	0.015812816
4662	B cell receptor signaling pathway	43	2158	48	2734	0.042316964
5211	Renal cell carcinoma	41	2158	45	2734	0.025499631
5220	Chronic myeloid leukemia	41	2158	45	2734	0.025499631
4115	p53 signaling pathway	39	2158	43	2734	0.034764924
5014	Amyotrophic lateral sclerosis (ALS)	37	2158	38	2734	0.001316211
5213	Endometrial cancer	32	2158	34	2734	0.015598239
520	Amino sugar and nucleotide sugar metabolism	26	2158	28	2734	0.046209141
51	Fructose and mannose metabolism	22	2158	23	2734	0.030443267
4330	Notch signaling pathway	20	2158	20	2734	0.008648864

^a^The number of significantly differentially expressed genes matching KEGG pathways.

^b^The total number of significantly differentially expressed genes.

^c^The number of genes matching KEGG pathways.

^d^The total number of genes.

### Validation of miRNA microarray data via Quantitative RT-PCR

**Figure 2:**
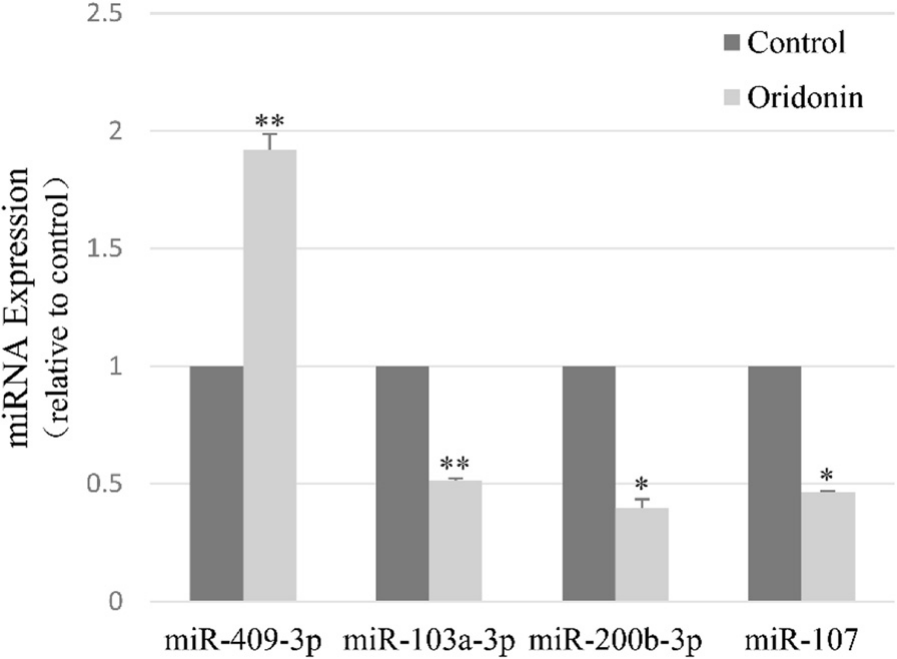
qPCR validation of a subset of miRNA microarray data. The horizontal axis represents the miRNAs, and the vertical axis represents the expression of miRNAs. The black bar represents the control group, and the grey bar represents the oridonin group. The data are expressed as the mean ± standard deviation (SD). **Significantly different from the control (p < 0.01); *different from the control (p < 0.05).

Among the significantly regulated miRNAs identified in the microarray assay, 4 miRNAs were selected for further validation via quantitative real-time PCR. The quantitative RT-PCR results showed that miR-409-3p was upregulated 2.04 times, miR-103a-3p was downregulated 1.85 times, miR-200b-3p was downregulated 2.22 times and miR-107 was downregulated 2.13 times in the oridonin treatment group compared with the control (Figure 2), which correlated well with the microarray results in Table 1.

## Discussion

With the discovery of miRNAs, it has been shown that miRNAs can function as endogenous posttranscriptional gene regulators through binding to the 3′ untranslated region of target mRNAs, and emerging evidence suggests that miRNAs play an important role in regulating diverse biological processes. Abnormal expression of miRNAs is associated with many diseases, such as nervous system diseases, cardiovascular disease and cancer. Several studies have demonstrated that aberrant miRNA expression is involved in pancreatic cancer (Table 2).

Pancreatic cancer is one of the most lethal malignancies, characterised by its highly metastatic potential, worst prognosis and strong resistance to chemotherapy and radiation therapy. The overall 5-year survival rate of pancreatic cancer is less than 5%. Chemotherapy and radiation therapy are the main therapeutic methods used to treat such cancers, however, these treatments produce deleterious side effects. Therefore, there is an urgent need to find safer treatments. Recently, traditional Chinese medicines have become a “hot spot” in relation to their potential anti-tumour properties, although the mechanisms of such anti-tumour effects are not clear. Some studies showed that the anti-cancer mechanisms of the active ingredients of traditional Chinese medicines may be associated with miRNAs, which can be treated as targets for cancer therapies [32-34]. Previous studies revealed that oridonin can cause cell cycle arrest, induce apoptosis and enhance the antitumour activity of gemcitabine in pancreatic cancer [35-37]. In this study, the miRNA expression was profiled in BxPC-3 human pancreatic cancer cells treated with oridonin. MicroRNA results showed that 105 miRNAs were significantly altered by oridonin treatment (Table 1). Among them, many have been reported to be associated with tumorigenesis or cancer progression. For instance, miR-424-5p (Table 3) is overexpressed in human pancreatic cancer. Down-regulation of miR-424-5p inhibits cell proliferation, migration and invasion and increases cell apoptosis in PANC-1 cells [22]. In addition, miR-17-5p, which is related to a poor prognosis, is overexpressed in pancreatic cancer [11]. Both miR-424-5p and miR-17-5p were found to be down-regulated by oridonin in our microarray data, implying that oridonin may inhibit pancreatic cancer cell proliferation, migration, invasion, and induce apoptosis by down-regulating miR-424-5p and miR-17-5p.

Four miRNAs (miR-409-3p, miR-103a-3p, miR-200b-3p and miR-107) were chosen to validate the microarray assay via quantitative real-time PCR. PCR results showed a well correlation with the microarray results, confirming the significant difference between oridonin treated and untreated cells. It has been reported that epigenetic silencing of miR-107 can regulate the expression of cyclin-dependent kinase 6 in pancreatic cancer [15]; while interfering miR-409-3p promotes tumour growth, the epithelial-to-mesenchymal transition (EMT) and bone metastasis [38]. miR-409-3p also suppresses the migration and invasion of bladder cancer T24 and 5,637 cells via targeting c-Met [39] and regulates cell proliferation and apoptosis by targeting PHF10 in SGC-7901 gastric cancer cells [40]. However, the effect of this miRNA on pancreatic cancer has rarely been described, similar to the situation for miR-103a-3p and miR-200b-3p. Based on the literature and our analysis on miRNA expression in cancer cells, we presume that these miRNAs likely play similar roles in pancreatic cancer, such as inhibiting cell proliferation, migration, invasion and inducing apoptosis. Thus, interruption of miRNA expression may be potential therapeutic targets for pancreatic cancer, although further studies are required to explore this possibility.

For further investigation, Gene Ontology analysis and KEGG pathway annotation were applied. GO enrichment analysis showed that the mRNA clusters were significantly enriched for the categories that are essential for cell survival. A total of 31 enrichment pathways for predicted target genes were listed in Table 4. Among them, the top 5 signaling pathways were neuroactive ligand-receptor interactions, Pathways in cancer, MAPK, focal adhesion and calcium signalling pathways. The results showed that 176 predicted target genes are associated with neuroactive ligand-receptor interactions, 138 genes are associated with MAPK signaling pathways, while 118 genes are associated with calcium signalling pathways. Data from previous research suggest that oridonin can enhance the antitumour activity of gemcitabine in pancreatic cancer through the MAPK-p38 signalling pathway [36] and inhibit BxPC-3 cell growth through caspase signaling pathways [41], which verified the results of KEGG pathway annotation. In conclusion, The KEGG pathway annotation revealed that BxPC-3 pancreatic cancer cells may be influenced by oridonin through these pathways and provided new research directions.

## Conclusion

In conclusion, the results of the present study provide new insights into the general mechanisms underlying the suppression of BxPC-3 cells by oridonin treatment and may provide new therapeutic methods for pancreatic cancer.
